# A novel link between Sus1 and the cytoplasmic mRNA decay machinery suggests a broad role in mRNA metabolism

**DOI:** 10.1186/1471-2121-11-19

**Published:** 2010-03-15

**Authors:** Bernardo Cuenca-Bono, Varinia García-Molinero, Pau Pascual-García, Encar García-Oliver, Ana Llopis, Susana Rodríguez-Navarro

**Affiliations:** 1 mRNA Transport Lab., Centro de Investigación Príncipe Felipe, E-46013, Valencia, Spain

## Abstract

**Background:**

Gene expression is achieved by the coordinated action of multiple factors to ensure a perfect synchrony from chromatin epigenetic regulation through to mRNA export. Sus1 is a conserved mRNA export/transcription factor and is a key player in coupling transcription initiation, elongation and mRNA export. In the nucleus, Sus1 is associated to the transcriptional co-activator SAGA and to the NPC associated complex termed TREX2/THSC. Through these associations, Sus1 mediates the nuclear dynamics of different gene loci and facilitate the export of the new transcripts.

**Results:**

In this study, we have investigated whether the yeast Sus1 protein is linked to factors involved in mRNA degradation pathways. We provide evidence for genetic interactions between *SUS1 *and genes coding for components of P-bodies such as *PAT1*, *LSM1*, *LSM6 *and *DHH1*. We demonstrate that *SUS1 *deletion is synthetic lethal with 5'→3' decay machinery components *LSM1 *and *PAT1 *and has a strong genetic interaction with *LSM6 *and *DHH1*. Interestingly, Sus1 overexpression led to an accumulation of Sus1 in cytoplasmic granules, which can co-localise with components of P-bodies and stress granules. In addition, we have identified novel physical interactions between Sus1 and factors associated to P-bodies/stress granules. Finally, absence of *LSM1 *and *PAT1 *slightly promotes the Sus1-TREX2 association.

**Conclusions:**

In this study, we found genetic and biochemical association between Sus1 and components responsible for cytoplasmic mRNA metabolism. Moreover, Sus1 accumulates in discrete cytoplasmic granules, which partially co-localise with P-bodies and stress granules under specific conditions. These interactions suggest a role for Sus1 in gene expression during cytoplasmic mRNA metabolism in addition to its nuclear function.

## Background

During gene expression, the coordinated action of several multiprotein complexes couple transcription, mRNA biogenesis and export, to guarantee the proper maturation of transcripts before their translation in the cytoplasm [1]. mRNA levels are highly regulated by transcription rate adjustments and mRNA decay, to produce the appropriate number of transcripts competent for translation [2]. In yeast, two major cytoplasmic mRNA degradation pathways control transcript turnover: the cytoplasmic exosome and the 5'→3' mRNA decay. Moreover, 5'→3' mRNA decay and translation are interconnected processes providing an exquisite equilibrium between degradation, storage and translation that correlates with the type and localisation of the mRNP in the cell (reviewed in [3]). Work over the last few years has shown that different classes of mRNPs are found as discrete granules in the cytoplasm. In yeast, different sorts of cytoplasmic mRNP granules have been described. Among them, P-bodies (PBs) and stress granules (SGs) are the best characterised (reviewed in [4,5]). P-bodies are implicated in translational repression, mRNA storage and 5'→3' mRNA decay [6]. The composition of PBs has been thoroughly studied. They are made up of a set of proteins that form the core of the particules, such as the decapping enzyme Dcp1/Dcp2, activators of decapping Dhh1, Pat1, Lsm1-7, Edc3 and the 5'→3' exonuclease Xrn1 [7]. Other proteins involved in different processes, such as nonsense-mediated decay (Upf1-3) [8] and translation (eIF4E, eIF4G and Pab1) [9,10] have also been reported to accumulate in these granules, but only under specific conditions.

A second class of well studied cytoplasmic mRNP structures are the stress granules (reviewed in [4]). SGs are cytoplasmic mRNP accumulations that appear when translation initiation is impaired. Study of stress granule formation has suggested that they contain mRNAs stalled in the process of translation initiation. In yeast they characteristically contain poly(A) mRNA, the poly(A)-binding protein Pab1, 40S ribosomal subunits and the translation factors eIF4E, eIF4G, eIF3 (reviewed in [4,5]).

In yeast but also in other organisms, both types of granules are interconnected (reviewed in [5]). Strikingly, assembly of stress granules depends on P-body formation and several factors are present in both granules, suggesting a crosstalk between them [6,10,11].

One key factor involved at different stages of nuclear mRNA metabolism is the conserved Sus1 protein, which is part of two stable nuclear complexes: the transcriptional coactivator SAGA and the nuclear pore associated TREX2 [12]. Biochemical and functional data have suggested a crucial nuclear role for Sus1 in coupling transcription activation and mRNA export. Previously, we have shown that Sus1 participates in histone H2B deubiquitination and histone H3 methylation together with the SAGA-DUB subunits Ubp8 and Sgf11 [13]. Sus1 mediates transcription activation through its associated with chromatin promoters as part of SAGA and is recruited to coding regions where it is necessary for transcription elongation [14]. Interestingly, Sus1 is also required for nuclear post-transcriptional events. After transcriptional shut off, Sus1 affects both the morphology as well as the persistent tethering of the mRNPs to their cognate gene, reinforcing the broad role of Sus1 in nuclear mRNA biogenesis [15]. Furthermore, Sus1 is crucial for TREX2-NPC interaction and its absence provokes a dramatic defect in mRNA export [12-14]. Altogether, Sus1 participates in many nuclear events from early epigenetic modifications to mRNA export through the nuclear pore (reviewed in [16]).

Strikingly, although Sus1's described functions take place in the nucleus it was also observed in the cytoplasm of yeast and *Drosophila *[14,17], thus suggesting additional roles outside of the nucleus.

In this study, we describe genetic and functional links between *SUS1 *and several components of P-bodies and stress granules. We demonstrate that *SUS1 *deletion is synthetic lethal with 5'→3' decay machinery components *LSM1 *and *PAT1 *and has a strong genetic interaction with *LSM6 *and *DHH1*. Interestingly, Sus1 overexpression leads to an accumulation of Sus1 at cytoplasmic granules, which can co-localise with P-bodies and stress granules. In addition, through affinity purification of TAP tagged Sus1, we have identified novel physical interactions between Sus1 and factors associated with P-body/stress granule. Finally, absence of *LSM1 *and *PAT1 *slightly promotes association between Sus1-TREX2. Taken together, our results reveal a novel link between the transcription/export factor Sus1 and cytoplasmic mRNA decay factors. Thus, Sus1 plays a broad role in mRNA metabolism.

## Results

### Sus1 interacts genetically with components of the mRNA degradation machinery

**Figure 1:**
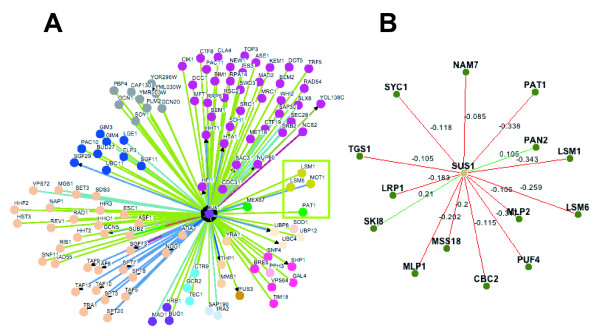
**Network showing Sus1 interactions**. (A) All known physical or genetic *SUS1 *interactions were downloaded from the BioGRID database [18]. Osprey software was used to obtain the graphical representation of the Sus1 network by gene ontology and a complete legend of colour settings can be found in [26]. (B) Network visualization of SGA genetic interactions involving genes that participate in mRNA processing. Positive SGA interactions are coloured in green, while negative SGA interactions are in red. A complete legend of colour setting and all information can be found in DRYGIN [20].

Unveiling the network of genetic interactions for a given factor provides clues to understand its role in a cellular context. To further uncover Sus1's molecular function, we utilized The BIOGRID database [18], searching for the complete genetic network of *sus1Δ*. Among others, we found a number of factors involved in mRNA biogenesis, whose deletion enhanced the *sus1Δ *growth defect (Figure 1A). Moreover, recent analysis of wide yeast genetic interactions confirms these data and extends the list of *sus1Δ *interactors involved in mRNA processing (Figure 1B) [19,20].

**Figure 2:**
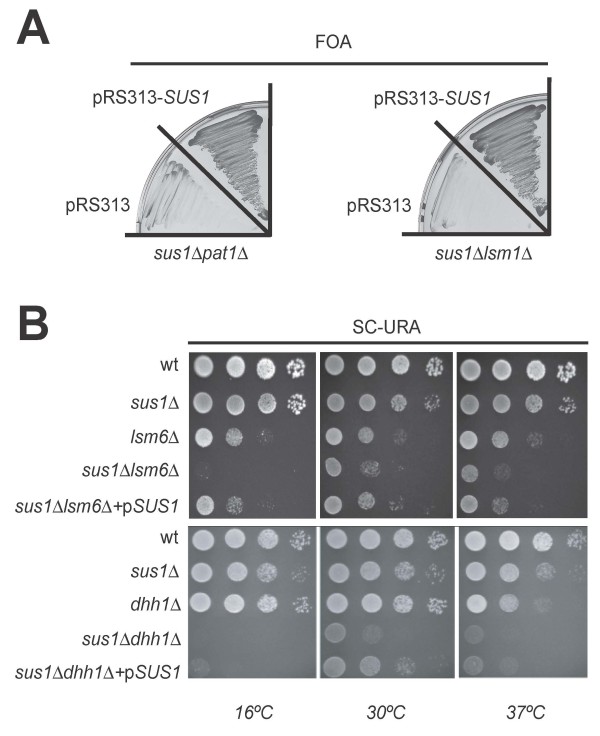
***SUS1 *Interacts Genetically with Genes Encoding Components of the mRNA decay machinery**. (A) *Synthetic lethality of sus1Δ with lsm1Δ and pat1Δ*. Double mutants containing a pRS316-*SUS1 *plasmid, were transformed with an empty vector (pRS313) or the same plasmid bearing a wild-type version of *SUS1 *(pRS313-*SUS1*). Transformants were streaked onto 5-fluoroorotic acid (FOA) containing plates, which were incubated at 30°C for 3 days. No growth indicates synthetic lethality. (B) *Synthetic sick phenotype of sus1Δ with lsm6Δ or dhh1Δ*. Wild-type (wt), single and double mutants were transformed with an empty vector (pRS316). The double mutants were also transformed with a pRS316-*SUS1 *(p*SUS1*) in order to complement the phenotype. Cells were diluted in 10^-1 ^steps, and equivalent amounts of cells were spotted on SC-URA plates.

To corroborate and extend this observation we generated double mutants of *SUS1 *combined with deletions in *PAT1*, *LSM1*, *LSM6 *or *DHH1 *and the resulting phenotypes were analysed. Deletion of either *PAT1 *or *LSM1 *in *sus1Δ *cells provokes a synthetic lethal phenotype (Figure 2A). In addition, absence of *LSM6 *or *DHH1 *elicits an enhancement of the slow growth associated with *sus1Δ *(Figure 2B). In conclusion, Sus1 genetically interacts with key components of the cytoplasmic 5'→3' mRNA decay machinery.

### When overexpressed, Sus1 accumulates at cytoplasmic granules

**Figure 3:**
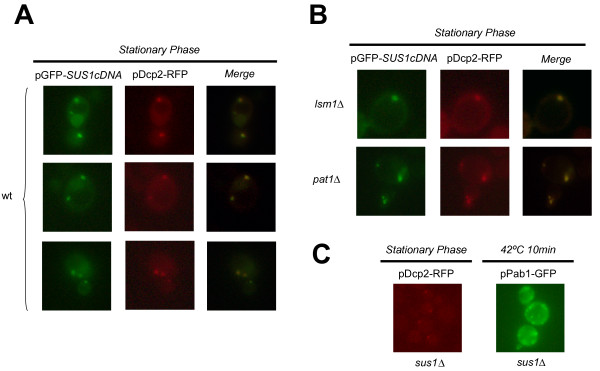
**Sus1 co-localises with P-bodies**. (A) *Sus1 partially localises at P-bodies in stationary phase*. Wild-type (wt) cells co-transformed with a plasmid containing the cDNA of *SUS1 *(pGFP-*SUS1cDNA*) and a plasmid containing Dcp2 (pDcp2-RFP) were observed in stationary phase. Partial co-localisation indicates localisation of Sus1 in P-bodies. Pictures were obtained by fluorescence microscope. (B) *Sus1 is present at P-bodies independently of Lsm1 or Pat1*. Cells expressing *SUS1cDNA *and Dcp2 in the different mutants were observed in stationary phase. Pictures were taken with a camera mounted onto a fluorescence microscope. (C) *SUS1 is not required for P-bodies or stress granules formation*. Cells expressing Dcp2-RFP or Pab1-GFP were transformed in *sus1Δ *and observed in stationary phase or after heat shock respectively. Images were generated using a fluorescence microscope.

Work from many laboratories has shown that some proteins involved in mRNA metabolism are constituents of mRNP cytoplasmic granules (reviewed in [4,5]). In light of the fact that there are genetic interactions between Sus1 and some of these factors, we asked whether Sus1 could accumulate at discrete cytoplasmic granules under specific conditions. Interestingly, after glucose starvation or in stationary phase, we observed an accumulation of Sus1 in discrete granules at the cytoplasm (data not shown). Moreover, overexpression of Sus1 from its cDNA (Cuenca-Bono et al., manuscript in preparation) enhanced this accumulation. Several types of cytoplasmic granules have been characterized in yeast including P-bodies and stress granules [5]. To address the nature of Sus1-containing granules, we co-localised the P-body component Dcp2 and Sus1 in cells expressing *SUS1 *cDNA. As shown in Figure 3A, Sus1 partially co-localises with P-bodies under these conditions. From this, we conclude that Sus1 can enter the P-bodies when overexpressed.

Different factors contribute to the assembly of P-bodies under glucose deprivation. The general decapping activators Dhh1, Pat1 and Lsm1 have different roles in P-body assembly and composition [21]. Since absence of Lsm1 or Pat1 is synthetic lethal with *sus1Δ*, we checked for the contribution of these factors to Sus1 presence at cytoplasmic granules. Cells lacking either *LSM1 *or *PAT1 *were transformed with pGFP-*SUS1cDNA *and pDcp2-RFP; and localisation analysis of both proteins by fluorescence microscopy was performed. As shown in Figure 3B, Sus1 and Dcp2 still co-localise in these mutant backgrounds. Therefore, we conclude that Sus1 co-localises with P-bodies independently of Lsm1 and Pat1. To analyze whether Sus1 might have a role in the assembly of cytoplasmic structures as P-bodies or stress granules, we localized Dcp2 or Pab1 in *sus1Δ *cells. As illustrated in Figure 3C Sus1 is dispensable for the formation of P-body and stress granule. Thus, we conclude that Sus1 appears not to be a structural component of P-bodies or stress granules.

### Sus1 interacts physically with components of P-bodies and stress granules

**Figure 4:**
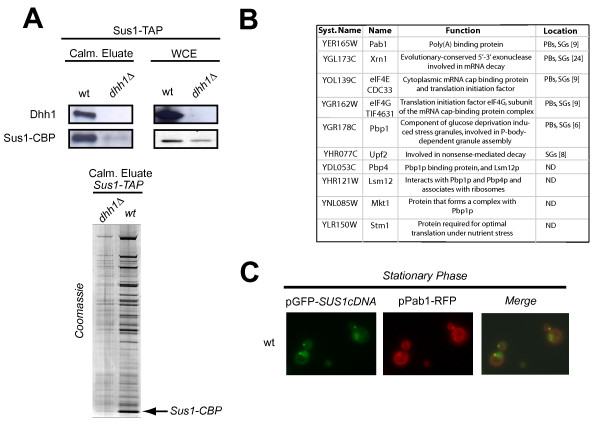
**Sus1 interacts with Dhh1 and co-localise with Pab1**. (A) *Sus1 physically interacts with Dhh1*. Sus1 co-purification with Dhh1 was revealed by western-blot of Sus1-TAP calmoduline eluates purificied from wild-type (wt), and *dhh1Δ *cells using anti-Dhh1 antibodies (left panel). Prior to purification, Sus1 and Dhh1 presences were confirmed by western blot analysis of whole cell extracts (WCE) (right panel). Lower panel shows the enriched calmoduline eluates from Sus1-TAP (wt), and Sus1-TAP*dhh1Δ *purifications analysed by SDS 4-12% gradient polyacrylamide gel electrophoresis stained with Coomassie (B) *MudPIT analysis of Sus1-TAP*. List of proteins co-purified with Sus1 identified by MudPIT. PBs (P-bodies); SGs (stress granules); ND (Not Determined) (C) *Sus1 is present at cytoplasmic Pab1-containing granules*. Sus1 co-localises with Pab1 in stationary phase.

Taken together our genetic and localisation results suggest that Sus1 could transiently enter P-bodies. A tentative idea is that Sus1 interacts with this mRNP early during transcription and a minor pool of the proteins could travel to the cytoplasm. To substantiate this idea, we studied whether Sus1 physically interacts with proteins found in P-bodies. Based on our genetic data we first tested the association between Sus1 and Dhh1. Cells expressing Sus1-TAP were grown in standard conditions and TAP purification was performed as described previously [12] and in Methods. As shown in Figure 4A, specific interaction between Sus1 and Dhh1 was demonstrated by western blot using anti-Dhh1 antibodies. Notably, we observed a reduction in Sus1 expression in whole cell extracts (WCE) of cells lacking *DHH1*. This decrease could impact on Sus1 stability since the profile of the enriched Sus1-TAP calmoduline eluate in *dhh1Δ *is drastically affected (Figure 4A lower panel). Hence, we conclude that Sus1 physically interacts with Dhh1 and loss of *DHH1 *affects Sus1 stability/expression.

To extend this observation, we performed Sus1-TAP purification and the enriched calmoduline eluate was this time analysed by multidimensional protein identification technology (MudPIT) in order to identify the polypeptide mixture present in our affinity purification. This kind of analysis has been extensively used to discover new interactors of a known protein and to study in more detail the proteomic characterization of different pathways [22]. Our MudPIT analysis (Figure 4B) reveals that besides SAGA and TREX2 subunits (data not shown), Sus1 co-purifies with the ribosome-associated factor Stm1 and the translation initiation factors eIF4E and eIF4G. Moreover, we also identified the poly(A) binding proteins Pab1, Pbp1, Pbp4, Lsm12 and Mkt1. Furthermore, peptides corresponding to the 5'→3' exonuclease Xrn1, present at PBs and SGs and Upf2 protein (involved in NMD and present at SGs) were identified in our purification. Altogether, our MudPIT analysis reinforces our genetic and localisation data thereby revealing new physical connections between Sus1 and factors involved in translation and mRNA metabolism, some of which are localised to P-bodies and/or stress granules.

To strengthen our biochemical result showing physical interaction between Sus1 and the SGs/PBs component Pab1, we checked for co-localisation between them in wild-type cells during stationary phase. As illustrated in Figure 4C, Sus1 and Pab1 co-localise at discrete cytoplasmic granules. Thus, we conclude that Sus1 is present in Pab1-containing granules, which support its presence at P-bodies and/or stress granules.

### mRNA decay components affect Sus1 protein interactions

**Figure 5:**
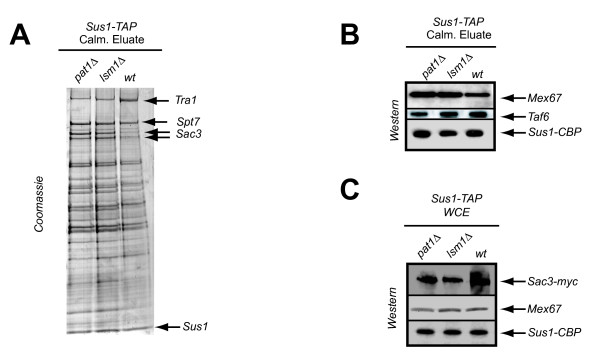
**Absence of the decay mutants partially enhances Sus1 binding to TREX2 and Mex67**. *Sus1 association to TREX2 is enhanced in pat1Δ *and *lsm1Δ*. Sus1-TAP was affinity-purified from wild-type (wt), *pat1Δ *and *lsm1Δ *strains. The enriched calmoduline eluates from all purifications were analysed by SDS 4-12% gradient polyacrylamide gel electrophoresis and proteins stained with Coomassie. Sus1, Tra1, Spt7 and Sac3 bands were verified by mass spectrometry. (B) *Sus1 association to Mex67 is increased in the absence of PAT1 or LSM1*. Enrichment of Mex67 in the calmoduline eluates from the respectives deletion strains is demonstrated by western blotting using anti-Mex67 antibodies, whereas no differences were observed for Taf6. (C) Sac3, Mex67 and Sus1 expression levels were confirmed by detecting similar loading from inputs by immunoblot.

Sus1 is part of two multiprotein assemblies, the SAGA and TREX2 complexes (reviewed in [16]). The levels of Sus1 binding to each complex is important for their correct functionality. The fact that *LSM1*, *PAT1 *and *DHH1 *are genetically linked to *SUS1*, prompted us to test whether Sus1 association with SAGA or TREX2 components was disrupted in the absence of these proteins. Hence, we genomically TAP-tagged Sus1 in *lsm1Δ *and *pat1Δ *strains and conducted Sus1-TAP purification using standard conditions. As illustrated in Figure 5A, Sus1 is associated with both SAGA and TREX2 in *lsm1Δ *and *pat1Δ *strains. However, comparison between the calmoduline eluates from the three purifications by coomassie staining revealed a reproducible enrichment of Sac3 (TREX2 subunit) in *lsm1Δ *and *pat1Δ *compared to the wild-type. We conclude that the absence of *LSM1 *and *PAT1 *improves Sus1-Sac3 association. Both Sac3 and Sus1 bind to the essential mRNA exporter Mex67. If loss of *LSM1 *and *PAT1 *promotes the interaction between Sus1 and TREX2 it may increase the association between Sus1 and Mex67. To test this possibility, we used western blot analysis to look for the presence of Mex67 in the calmoduline eluates from our purifications. As shown in Figure 5B a slight increase of the associated mRNA export factor Mex67 is observed, whereas no enrichment of the SAGA subunit Taf6 was found. As loading controls, western blots of whole cell extracts (WCE) revealed similar expression for these proteins in wild-type and in both mutants strains (Figure 5C). Taken together, these results suggest that loss of Lsm1 or Pat1 alters the kinetics of Sus1 associations.

## Discussion

Sus1 is genetically linked to proteins involved in different stages of gene expression. Previously, we reported that Sus1 deletion is synthetic lethal with deletions in mRNA export/biogenesis factors, namely Mex67, Yra1, Dbp5, Nab2 and Sub2 [12]. In this study we show strong genetic interactions between *SUS1 *and *PAT1 *or *LSM1 *(synthetic lethality), and *DHH1 *or *LSM6 *(synthetic enhancement). Lsm1, Lsm6, Pat1 and Dhh1 are proteins involved in mRNA degradation that are localised at cytoplasmic P-bodies [21]. Thus these new genetic interactions between Sus1 and these factors suggest a connection between Sus1 and cytoplasmic mRNA metabolism.

To gain knowledge about the functional meaning of these genetic interactions, we have investigated the presence of Sus1 at P-bodies. In addition to the genetic link between Sus1 and P-body components, we have been able to localise Sus1 at cytoplasmic granules, by showing that it partially co-localises with Dcp2 and Pab1. Moreover, Sus1-Dcp2 co-localisation in the cytoplasmic granules is independent of Lsm1 or Pat1 and we have demonstrated that Sus1 is dispensable for PB or SG assembly.

We showed previously that under standard growth conditions Sus1-GFP is localized at the NPC and the nuclear lumen, but that a weak signal could be detected in the cytoplasm [12]. However, Sus1 was not visible at these granules suggesting that Sus1 co-localisation with P-bodies might be very dynamic and transient. Accordingly, we have shown that Sus1 co-purifies with P-body components Dhh1 and Xrn1, after standard Sus1-TAP purification. Thus it is likely that a minor pool of the protein, undetectable by our fluorescence analysis, is in fact associated with cytoplasmic structures in physiological conditions. Strikingly, during the revision process of this work, *Drosophila *Sus1 (ENY2) has been observed in the cytoplasm [17]. Kopytova and coworkers detected a significant amount of ENY2 in the cytoplasm of *Drosophila *S2 cells and they conclude that ENY2 may also be of significance for the fate of mRNPs in the cytoplasm.

Sus1 also co-purifies with factors involved in translation initiation and poly(A) binding proteins, which have also been reported to enter P-bodies and to be constituents of stress granules [8-10,23]. By MudPIT analysis of our standard Sus1-TAP purification, peptides for eIF4E, eIF4G, Pab1, Pbp1, Pbp4, Lsm12, Mkt1 and Stm1 were found. In glucose starvation conditions, eIF4E, eIF4G and Pab1 were shown to keep away from ribosomes and to localise at cytoplasmic granules originally named EGP-bodies (eIF4**E**, eIF4**G**, **P**ab1) [9]. Observations by Hoyle and co-workers demonstrate that these mediators accumulate both in P-bodies and in EGP-bodies. In these lines, co-localisation of Sus1 and Pab1 demonstrates that Sus1 is present in Pab1-containing granules during stationary phase, which supports our previous data and opens the possibility of Sus1 presence also at SG and/or EGP-bodies. In addition, Sus1 also interacts with Stm1, which promotes Dhh1 function in translation repression and mRNA decay [24].

What is Sus1 function in the cytoplasmic mRNA cycle? We speculate that Sus1 can travel with the mRNPs, especially since it is loaded early during transcription. It may then facilitate the interaction with other factors thereby providing a way to connect transcription and translation through mRNP metabolism. A possible scenario is that Sus1 mediates different physical interactions between the P-body machinery and translation, to contribute to the fate of the mRNA in the cytoplasm. In support of this model, the resolution of the Sus1 structure in association with TREX2 or SAGA has revealed that its structure is compatible with multiple associations [25]. The extended fold of Sus1 creates a surprisingly large surface area for a protein of this size and this could facilitate the association of Sus1 with other factors. Previous work suggested that eIF4E, eIF4G and Pab1 could be present on a subpopulation of mRNAs in the P-bodies [10]. It was suggested that such mRNA subpopulations could be a specific set of transcripts. Based on these arguments it is possible that Sus1 contributes to the mRNA metabolism of SAGA dependent genes in the cytoplasm as well as in the nucleus. Further experiments to characterize the composition of Sus1 cytoplasmic structures will help us to verify this hypothesis. Remarkably, a possible cytoplasmic role for Sus1 is likely to be conserved through evolution since ENY2 is present in the cytoplasm of *Drosophila *S2 cells [17], a finding which strongly supports our data. Future work will help us to reveal new insights in to how Sus1 participates in gene expression from chromatin modifications to mRNA metabolism in the cytoplasm.

## Conclusions

Sus1 is genetically linked to factors involved in mRNA decay. Sus1 is observed at P-bodies and stress granules when overexpressed and it interacts physically with components of these cytoplasmic structures in normal conditions.

## Methods

### Yeast Strains, Plasmids, Microbiological Work

Yeast strains and plasmids used in this study are listed in Additional file 1 (Supplemental Table 1 and Supplemental Table 2, respectively). Microbiological techniques, yeast plasmid transformation, mating, sporulation of diploids and tetrad analysis were done essentially as described previously [14]. For spotting analyses, cells were grown on synthetic selective medium (Synthetic complete medium: glucose 2%, ammonium sulphate 0,4%, yeast nitrogen base 0,34%, and supplements (Dropout)) to 0.5 OD_600 _and subjected to 10-fold serial dilutions. Chromosomal integration of TAP and MYC as C-terminal tags were performed as described in [14]. Genetic interaction studies were performed by growing the double mutants under appropriated conditions. An empty plasmid (pRS313) and the same plasmid containing *SUS1 *were used for synthetic lethal assays. The dotspot assays were performed by transforming the cells with an empty plasmid (pRS316) and the same plasmid backbone bearing *SUS1 *for complementation.

### TAP Purifications, MudPIT and Western Blot Analysis

TAP purifications of wild-type (BY4741) and mutant strains were performed as described previously [12]. Sus1-TAP fusion protein and associated proteins were recovered from cell extracts by affinity selection on an IgG matrix. After washing, the TEV protease is added to release the bound material. The eluate is incubated with calmodulin-coated beads in the presence of calcium. This second affinity step is required to remove the TEV protease as well as traces of contaminants remaining after the first affinity selection. After washing, the bound material is released with EGTA. This enriched fraction is called Calmoduline eluate. Calmoduline eluates from the TAP-purified complexes were analyzed by SDS-PAGE by using Novex 4-12% gradient gels (Invitrogen) and visualized by staining with Novex Colloidal Blue staining kit (Invitrogen). MudPIT analyses were performed by mass spectrometry as described in [22]. Western blot analysis was performed using anti-Mex67, anti-Taf6, anti-Dhh1, anti-MYC and anti-TAP according to standard procedures.

### Preparation of Cells for Fluorescence Microscopy

For observation at stationary phase, cells were grown for two days in synthetic selective medium supplemented with amino acids. Cells were then washed and resuspended in fresh media prior to observation. For observation at 42°C, cells were treated for 10 minutes at 42°C and immediately observed. Pictures were made using a Leica DM6000B fluorescence microscope.

### Sus1 Network Representation

Data available from the BIOGRID [18] were downloaded and used to represent Sus1 interactions with the Osprey 1.2.0 software available at [18]. A complete legend of colour settings can be found in [18]. Network visualization of SGA genetic interactions involving genes that participate in mRNA processing were downloaded from DRYGIN database. A complete legend of colour settings and all information can be found in [18].

## List of abbreviations

mRNA: messenger RiboNucleic Acid; SAGA: Spt-Ada-Gcn5-acetyltransferase. NPC: Nuclear Pore Complex; TREX2/THSC: Sac3-Thp1-Cdc31-Sus1 complex; P-bodies/PBs: Processing bodies; SGs: Stress Granules; mRNP: messenger RiboNucleoProtein; Poly(A): Polyadenilated mRNA tail; SAGA-DUB: SAGA DeUbiquitination Module; MudPIT: Multidimensional Protein Identification Technology; NMD: Nonsense Mediated Decay; EGP-bodies: eIF4E, eIF4G and Pab1p bodies.

## Authors' contributions

BCB participates in the design of the study, carried out the molecular genetic studies, microscopy work and helped to draft the manuscript. VGM contributed to the microscope work, genetic studies and strain construction. PPG carried out the biochemical analysis and strains construction. EGO participates in the TAP purification and western blot analysis. ALL assisted technically in all parts of the work. SRN conceived the study, participated in microscope and biochemical analysis, coordinated the work and wrote the paper. All authors read and approved the final manuscript.

## Supplementary Material

Additional file 1 **Supplementary tables**. Yeast strains and plasmids used in this study. Click here for file
