# Supplementary material for: A novel link between Sus1 and the cytoplasmic mRNA decay machinery suggests a broad role in mRNA metabolism
Source: BMC Cell Biol. 2010 Mar 15;11:19. doi: 10.1186/1471-2121-11-19 (PMC2848209; doi:10.1186/1471-2121-11-19)
Supplement: Additional file 1 — Supplementary tables. Yeast strains and plasmids used in this study. [file 1471-2121-11-19-S1.PDF]

Supplementary Table 1 Cuenca-Bono et al., 2010

Yeast strain	Genotype	Reference
BY4741	Mat a, leu2-Δ0, his3-Δ1, met15-Δ0, ura3-Δ0	EUROSCARF
lsm1Δ	Mat a, leu2-Δ0, his3-Δ1, met15-Δ0, ura3-Δ0, lsm1::KanMX4	EUROSCARF
lsm6Δ	Mat a, leu2-Δ0, his3-Δ1, met15-Δ0, ura3-Δ0, lsm6::KanMX4	EUROSCARF
pat1Δ	Mat a, leu2-Δ0, his3-Δ1, met15-Δ0, ura3-Δ0, pat1::KanMX4	EUROSCARF
dhh1Δ	Mat a, leu2-Δ0, his3-Δ1, met15-Δ0, ura3-Δ0, dhh1::KanMX4	EUROSCARF
sus1Δ	Mat α, his3-Δ1, leu2-Δ0, lys2-Δ0, ura3-Δ0, sus1::KanMX4	EUROSCARF
lsm1Δ sus1Δ	Mat a, leu2-Δ0, his3-Δ1, met15-Δ0, ura3-Δ0, lsm1::KanMX4, sus1::KanMX4	This study
lsm6Δ sus1Δ	Mat a, leu2-Δ0, his3-Δ1, met15-Δ0, ura3-Δ0, lsm6::KanMX4, sus1::KanMX4	This study
pat1Δ sus1Δ	Mat a, leu2-Δ0, his3-Δ1, met15-Δ0, ura3-Δ0, pat1::KanMX4, sus1::KanMX4	This study
dhh1Δ sus1Δ	Mat a, leu2-Δ0, his3-Δ1, met15-Δ0, ura3-Δ0, dhh1::KanMX4, sus1::KanMX4	This study
SUS1-TAP	Mat α, ade2, his3, leu2, trp1, ura3, SUS1-TAP::TRP1	[1]
lsm1Δ SUS1-TAP	Mat a, leu2-Δ0, his3-Δ1, met15-Δ0, ura3-Δ0, lsm1::KanMX4, SUS1-TAP::URA	This study
pat1Δ SUS1-TAP	Mat a, leu2-Δ0, his3-Δ1, met15-Δ0, ura3-Δ0, pat1::KanMX4, SUS1-TAP::URA	This study
dhh1Δ SUS1-TAP	Mat a, leu2-Δ0, his3-Δ1, met15-Δ0, ura3-Δ0, dhh1::KanMX4, SUS1-TAP::URA	This study
SUS1-TAP SAC3-MYC	Mat a, leu2-Δ0, his3-Δ1, met15-Δ0, ura3-Δ0, SUS1-TAP::URA, SAC3-MYC::HIS	This study
lsm1Δ SUS1-TAP SAC3-MYC	Mat a, leu2-Δ0, his3-Δ1, met15-Δ0, ura3-Δ0, lsm1::KanMX4, SUS1-TAP::URA, SAC3-MYC::HIS	This study
pat1Δ SUS1-TAP SAC3-MYC	Mat a, leu2-Δ0, his3-Δ1, met15-Δ0, ura3-Δ0, pat1::KanMX4, SUS1-TAP::URA, SAC3-MYC::HIS	This study

Supplementary Table 2 Cuenca-Bono et al., 2010

Name	Description	Source
pNOPGFP1L	*GFP, LEU2, CEN*	[2]
pGFP-*SUS1*	*GFP-SUS1, LEU2, CEN* (based in pNOPGFP1L)	This study
pGFP-*SUS1cDNA*	*GFP-SUS1cDNA, LEU2, CEN* (based in pNOPGFP1L)	This study
pRS313	*HIS3, CEN*	[3]
pRS313-*SUS1*	*SUS1, HIS3, CEN*	This study
pRS316	*URA3, CEN*	[3]
pRS316-*SUS1* (p*SUS1*)	*SUS1, URA3, CEN*	This study
pDcp2-RFP	*DCP2-RFP, URA3, CEN*	[4]
pPab1-RFP	*PAB1-RFP, URA3, CEN*	[5]
pPab1-GFP	*PAB1-GFP, URA3, CEN*	[5]

Literature Cited for Supplemental Tables:

[1] Rodriguez-Navarro, S. et al. (2004). Sus1, a functional component of the SAGA histone acetylase complex and the nuclear pore-associated mRNA export machinery. *Cell* 116, 75-86.

[2] Hellmuth, K., Lau, D.M., Bischoff, F.R., Kunzler, M., Hurt, E. and Simos, G. (1998). Yeast Los1p has properties of an exportin-like nucleocytoplasmic transport factor for tRNA. *Mol. Cell Biol.* 18, 6374-86.

[3] Sikorski, R.S. and Hieter, P. (1989). A system of shuttle vectors and yeast host strains designed for efficient manipulation of DNA in Saccharomyces cerevisiae. *Genetics* 122, 19-27.

[4] Brengues, M. and Parker, R. (2007). Accumulation of polyadenylated mRNA, Pab1p, eIF4E, and eIF4G with P-bodies in Saccharomyces cerevisiae. *Mol. Biol. Cell* 18, 2592-602.

[5] Scarcelli, J.J., Viggiano, S., Hodge, C.A., Heath, C.V., Amberg, D.C. and Cole, C.N. (2008). Synthetic genetic array analysis in Saccharomyces cerevisiae provides evidence for an interaction between RAT8/DBP5 and genes encoding P-body components. *Genetics* 179, 1945-55.
